# Effect of Moisture on Polymer Deconstruction in HCl Gas Hydrolysis of Wood

**DOI:** 10.1021/acsomega.1c06773

**Published:** 2022-02-16

**Authors:** Tainise Lourençon, Michael Altgen, Timo Pääkkönen, Valentina Guccini, Paavo Penttilä, Eero Kontturi, Lauri Rautkari

**Affiliations:** † Department of Bioproducts and Biosystems, Aalto University, P.O. Box 16300, FI-00076 Aalto, Espoo, Finland; ‡ Department of Biology, Institute of Wood Science, Universität Hamburg, Leuschnerstraße 91c, DE-21031 Hamburg, Germany

## Abstract

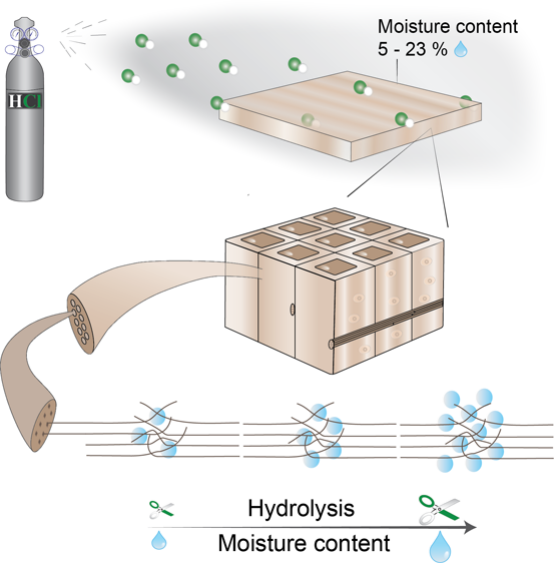

The HCl gas system previously used to produce cellulose nanocrystals was applied on Scots pine wood, aiming at a controlled deconstruction of its macrostructure while understanding the effect on its microstructure. The HCl gas treatments resulted in a well-preserved cellular structure of the wood. Differences in wood initial moisture content (iMC) prior to HCl gas treatment played a key role in hydrolysis rather than the studied range of exposure time to the acidic gas. Higher iMCs were correlated with a higher degradation of hemicellulose, while crystalline cellulose microfibrils were not largely affected by the treatments. Remarkably, the hydrogen–deuterium exchange technique showed an increase in accessible OH group concentration at higher iMCs, despite the additional loss in hemicelluloses. Unrelated to changes in the accessible OH group concentration, the HCl gas treatment reduced the concentration of absorbed D_2_O molecules.

## Introduction

In the urge to exit the fossil fuel era, many advances in materials science are bringing up methods to enable a full or controlled deconstruction of plant biomasses to construct functional, renewable, smart, and new bioproducts. Cellulose, lignin, and hemicellulose are found in the cell wall of plants and have shown to be great sources for the development of materials spanning from pharmaceutical to aerospace applications.^1−4^ Plant biomass can be broken down to a colloidal form, such as nanocellulose or colloidal lignin, or bigger particles, such as pulp fibers that make use of its hierarchical structure. Wood is one of the most abundant and economically important sources of biomass in the creation of new bioproducts. Although wood-based materials have played an important role for centuries, traditional wood end-products as furniture and pulp and paper are expanding, throughout new technologies, into multifunctional, smart materials as the example of transparent and elastic wood.^5,6^

The magnitude of biomass deconstruction depends on the applied process. In chemical approaches, often a liquid-phase system will penetrate the biomass. The main aspect influencing such deconstruction is accessibility, that is, the porosity and permeability of the material, which are related to void spaces in a solid and the ability with which a liquid can be transported, respectively.^7^ The methods to partially or fully deconstruct solid biomass such as wood are aggressive approaches in which water has a key role. Common methods like soda or kraft pulping or milder hydrothermal processes require a long mass transfer path to deconstruct the complex macrostructure of wood. In addition, a subsequent treatment for the full separation of components and the recovery of water is essential.

Acid methods are common approaches to break down the biomass. Aqueous acid hydrolysis is commonly applied either for the quantification of sugars in wood material^8^ or as pretreatment for enzymatic hydrolysis aiming for the recovery of sugars.^9^ It is also the main method for producing cellulose nanocrystals (CNCs).^10^ On the other hand, studies in the 1960–80s progressed with HCl gas hydrolysis aiming at high yields of polysaccharides.^11−14^ Our group recently showed an efficient HCl gas-based procedure^15^ applied to produce CNCs. The benefit of gas over aqueous and vapor systems, besides high yields of CNCs, lies in the higher accessibility of the substrate to the gas molecules and the lower volumes of water to recover. In addition, room temperature conditions are sufficient to reach the appropriate level of hydrolysis to CNCs. However, several open questions remain related to the factors controlling the reactivity of different cellulosic substrates to HCl gas hydrolysis. Especially the role of initial moisture content is of high interest because the dissociation of the HCl molecules and the hydrolysis reaction itself require water.^15,16^ At the same time, the nanoscale structure and its accessibility in many cellulosic materials are highly sensitive to moisture changes.

It is known that the HCl gas and vapor hydrolysis modifies the structure of cellulosic substrates at the level of cellulose microfibrils. Especially, studies on filter paper,^16^ bacterial cellulose,^17^ and hardwood cellulose nanofibers^18^ have reported increases in cellulose crystallinity with minimal effects on the crystallite dimensions. This effect has been explained by the crystallization of cleaved cellulose chains either in the small disordered domains along the microfibrils^19^ or on the hydrophilic surfaces of the cellulose crystals,^20^ but the results are not fully consistent. In another work, the HCl vapor hydrolysis of cotton filter papers was found to decrease the water-holding capacity of the cell walls, which was at least partly related to tighter aggregation of cellulose microfibrils in the hydrolyzed samples.^21^ Although these studies have outlined the structural changes caused by the hydrolysis in various substrates, its effect on the microfibril structures in unmodified wood still remains unexplored.

HCl gas systems have been proven to be effective both as pretreatment for polysaccharides^12^ and CNC production.^15^ However, to the best of our knowledge, there is no evidence of an HCl gas system applied for controlled partial deconstruction of wood. The same advantages found with HCl gas for CNC production, regarding less water to recover and higher mobility of gas compared to vapor, are expected for the wood substrate. We investigate herein, the manipulation of the supramolecular structure of bulky wood material to gain a deeper understanding of chemical changes that resulted in its complex microstructure after HCl gas treatment. In particular, we tested the role of moisture on the effects of the HCl gas treatment by conditioning wood blocks to different initial moisture contents (iMCs) prior to the treatment. Subsequently, the HCl gas-treated samples were thoroughly analyzed in terms of visual aspects, chemical structure, sorption behavior, and crystallinity.

We identified that increment in iMC played an important role in the degree of hydrolysis. Our findings extend to the contradictory observations on enhancement of OH accessibility despite the additional loss in hemicelluloses and, unrelated to OH accessibility, a decreased absorption of heavy water after the HCl treatments.

## Materials and Methods

### Materials

HCl gas (99.8%, 10 dm^3^, 6 kg) was purchased from AGA (Sweden). Aqueous NaOH (50%), diluted to neutralize acid gas residues, was purchased from AKA Chemicals, Finland. *N*,*N*-Dimethylacetamide (DMAc) was purchased from VWR Chemicals (EC) and LiCl from Merck (Darmstadt, Germany).

Kiln-dried boards of Scots pine (*Pinus sylvestris* L.) were used to prepare samples with dimensions of 20 × 20 × 5 mm^3^ (radial × tangential × longitudinal). The samples were free of heartwood and visible defects. All samples were vacuum-impregnated with acetone (ca. 1 h at 400 mbar) and extracted for 6 h in a Soxhlet apparatus. After extraction, they were kept under a fume hood overnight, which was followed by oven-drying at 103 °C for ca. 24 h to determine the initial dry mass and dimensions. Prior to the HCl gas treatment, the samples were placed in desiccators over saturated, aqueous solutions of either CaCl_2_, NH_4_Cl, or deionized water, which generated relative humidities (RHs) of ca. 33, 79, and >97%, respectively, at 20 °C.^22−24^ The samples with an approximate dry sample mass of 0.8–0.9 g were stored within the desiccators until their mass change was less than 0.1% within ca. 24 h, which was determined at a resolution of 0.001 g. The conditioning of the samples at 33, 79, and >97% RH resulted in iMCs of 5.1, 13.6, and 22.9%, respectively.

### Hydrogen Chloride Gas Hydrolysis

**Figure 1:**
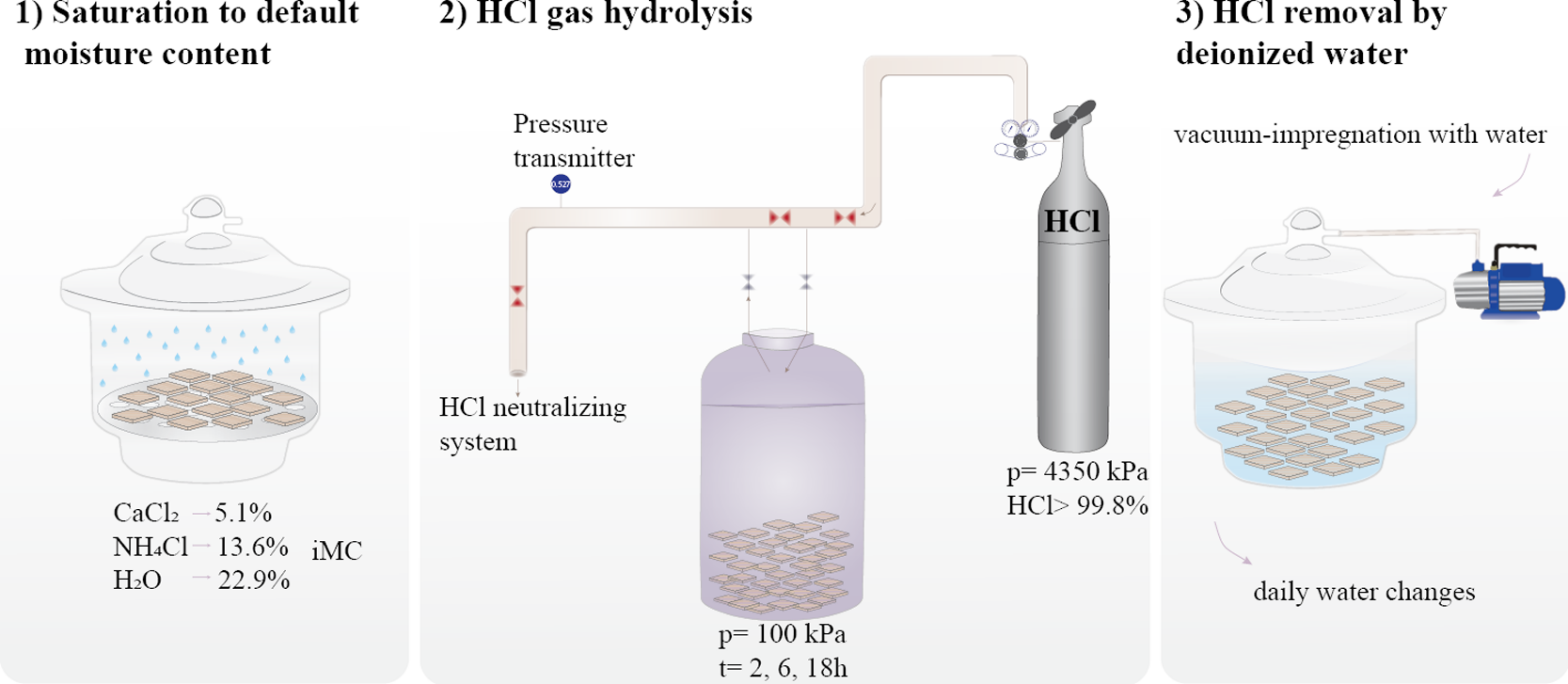
Simplified schematic picture of the HCl gas hydrolysis process for kiln-dried boards of Scots pine. First, pine boards were stored in desiccators until default moisture content was reached (1). Then, pine boards were loaded to the glass reactor, and the system was closed prior to the addition of 100 kPa HCl gas pressure from gas bottle (43 bar pressure). Gas pressure was released to the neutralizing system after acid hydrolysis (2). Finally, pine boards were washed with deionized water to remove the hydrolyzed products and remaining acid (3).

HCl gas hydrolysis was conducted with the samples at iMCs of 5.1, 13.6, and 22.9%. For each initial moisture level, 10 samples were acid-hydrolyzed with 3 different hydrolysis times: 2, 6, or 18 h. The samples were added to a Duran pressure plus^+^ glass bottle (volume 1 dm^3^) and hydrolyzed at 1 bar HCl gas pressure with a custom-built HCl gas reactor.^15^ After exposure to HCl gas, the samples were immediately placed into deionized water and vacuum-impregnated with water at ca. 50 mbar for ca. 1 h on the same day. The samples were stored in water with daily water changes for 2 weeks to remove the remaining HCl. At the end of the water-soaking treatment, the pH of the water did not decrease below pH 5 within 24 h for any of the sample groups (Figure 1).

### Changes in Sample Mass and Dimensions

Wet dimensions were recorded at the end of the water-soaking treatment. The samples were then dried at a temperature sequence of 20, 40, and 103 °C, with each step being held for ca. 24 h, to determine the dry mass and dry dimensions. This was followed by vacuum-impregnation with deionized water at 50 mbar for ca. 1 h and soaking the samples in water for ca. 24 h to record the wet dimensions after resoaking. Relative dimensions were calculated by relating the cross-sectional area (radial × tangential) of each sample in either wet, dry, or resoaked state to its initial dry cross-sectional area prior to the HCl gas treatment. Correspondingly, the relative dry mass was calculated by relating the dry mass after the HCl gas treatment to the initial dry mass.

### Chemical Composition Analysis

Three samples per sample group (iMC and treatment duration) were milled in a Wiley mill to pass through a 30 mesh screen. The particles were extracted in a Soxhlet apparatus with acetone for 6 h. Lignin and carbohydrates were determined by acid hydrolysis according to NREL/TP-510-42618.^8^ Carbohydrates were determined by high-performance anion-exchange chromatography with pulsed amperometric detection (HPAEC-PAD) in a Dionex ICS-3000 column. The acid-insoluble lignin content was determined gravimetrically as the acid-insoluble fraction after drying at 103 °C for 12 h. The acid-soluble lignin content was determined in a Shimadzu UV-2550 spectrophotometer using a wavelength of 205 nm and an absorptivity constant of 110 L g^–1^ cm^–1^. The total lignin content was calculated as the sum of the acid-soluble and -insoluble fraction. The ash content was determined according to TAPPI 211 om-02 by exposing oven-dried samples to 525 °C for 5 h. The chemical composition was first calculated on an as-received, oven-dry basis. This was corrected for the oven-dry mass loss (ML, in %) caused by the HCl gas treatment using a correction factor of (100 – ML)/100. Thereby, the chemical composition was related to the initial dry mass.

### Scanning Electron Microscopy

The morphology of the wood samples was analyzed using a field emission scanning electron microscope (Zeiss Sigma VP, Germany) using an acceleration voltage of 1.6 kV and a working distance of ca. 4.5 mm. Smooth surfaces of the wood samples were created using water-soaked samples and a rotary microtome. The samples were sputtered using Au–Pd targets to a thickness of ca. 4 nm.

### Dynamic Water Vapor Sorption

Sorption isotherms were measured on milled wood particles using a dynamic vapor sorption (DVS) apparatus (DVS intrinsic, Surface Measurement Systems, UK). Besides reference samples, only samples that were treated in HCl gas for 18 h were analyzed. Throughout the measurements, temperature and nitrogen flow were kept constant at 25 °C and 200 sccm, respectively. For each measurement, ca. 20 mg of wood particles were placed on the sample pan of the DVS apparatus and exposed to the following RH sequence: 0, 5, 15, 25, 35, 45, 55, 65, 75, 85, and 95%, and this was followed by a decrease of the RH in the reverse order. Two full cycles of absorption and (scanning) desorption were applied, but only the second sorption cycle was used to study the sorption behavior of the wood because sorption isotherms obtained from the first sorption cycle are sometimes not reproducible for treated woods.^25−27^ Each RH step was held until the sample mass change per minute (d*m*/d*t*) was less than 0.001% min^–1^ for a minimum of 10 min. This d*m*/d*t* was calculated using a regression window of 10 min. The mass at the end of each step was used to calculate the moisture content (MC) as the mass of absorbed water per wood dry mass (in %). Furthermore, the moisture content ratio was calculated for each RH set by relating the MC of the treated sample to the corresponding MC of the reference sample.

### Theoretical OH Accessibility

From the chemical composition data (extractive-free, as-received basis), the concentrations of cellulose and hemicelluloses were determined.^28^ Based on the explanations of Thybring et al. (2017),^29^ we assumed OH group contents of 18.5, 16.7–17.2, and 7.5–9.2 mmol g^–1^ for cellulose, hemicelluloses, and lignin, respectively. Furthermore, the cellulose was assumed to have an OH accessibility of 21%. Thybring et al. (2017)^29^ calculated this number by considering the accessible OH groups capable of deuterium exchange on surface cellulose chains in different cellulose microfibril geometries. They calculated hydroxyl accessibilities between 14.8 and 24.0% for seven different cellulose models, with 21% being the average. Using these constants and the measured chemical composition data, the lower and upper limits of the theoretical accessible OH group content were calculated for each sample group.

### Hydrogen–Deuterium Exchange

The accessible OH group content was determined by the hydrogen–deuterium exchange approach, as described previously.^30^ In short, ca. 15 mg of wood particles was placed on the sample pan of the DVS apparatus (DVS ET, Surface Measurement Systems, UK). The sample was first dried at 60 °C for 6 h using a preheater, which was followed by a decrease in the temperature to 25 °C and a temperature stabilization period of 2 h. Hydrogen–deuterium exchange was then induced by exposing the sample to D_2_O vapor at a target RH of 95% for 12 h. Finally, the sample was dried as described above. The accessible OH group content and the amount of absorbed D_2_O during the exchange step (both in mmol g^–1^) were calculated using the equations given by Altgen et al. (2020).^30^

### Molar Mass Distribution

Wiley milled samples (30 mesh) were extracted with acetone in a Soxhlet apparatus for 6 h. Peracetic acid (PAA) treatment (45 min, 85 °C) was repeated 4 times to convert wood powders to holocellulose. The molar mass distribution of wood holocellulose samples was determined by gel permeation liquid chromatography (GPC). First, the samples were activated by a water–acetone–DMAc sequence. Then, the activated samples were dissolved in 90 g/L LiCl containing DMAc at room temperature and under gentle stirring. The samples were then diluted to 9 g/L LiCl/DMAc, filtered with 0.2 μm syringe filters, and fed to a Dionex Ultimate 3000 system equipped with four PLgel MIXED-A 7.5 × 300 mm columns and refractive index (RI) detector Shodex RI-101. LiCl/DMAc was used as the eluent. Pullulan standards (343 Da–708 kDa, Polymer Standard Service GmbH, Germany, and 1600 kDa, Fluka GmbH, Germany) were used as calibrants. The molar masses of pullulan standards were converted to correspond to those of cellulose, using the equation *M*_cellulose_ = *q* × (*M*_Pullulan_)^*p*^.^31^

### X-ray Scattering

Wide-angle X-ray scattering (WAXS) data were recorded using a Xeuss 3.0 C (Xenocs, France) scattering device, with a GeniX 3D microfocus Cu source (wavelength λ = 1.54 Å) and an EIGER2 R 1M hybrid pixel detector set at 60 mm distance from the sample. By using the virtual-detector mode with three images (exposure time 600 s each) recorded in the horizontal plane, a *q* range of 0.09–4.2 Å^–1^ in the horizontal plane and 0.09–2.8 Å^–1^ in the vertical plane could be covered. The scattering vector *q* is defined as *q* = 4πsin(θ)/λ with scattering angle 2θ. Tangential-longitudinal wood sections with a thickness of approximately 200 μm were attached to a sample holder for solid samples with the fiber direction approximately vertical, and the measurement was carried out in a vacuum to avoid scattering from the air. The two-dimensional scattering images were corrected for cosmic background and normalized by the transmitted intensity, and the scattering from an empty sample holder was subtracted. The intensities were azimuthally averaged over 20° wide sectors around the equatorial and meridional intensity maxima. The lattice spacing and crystallite width perpendicular to the (200) planes of cellulose I_β_ were analyzed from the equatorial intensity profile and the lattice spacing and crystallite length perpendicular to the (004) planes from the meridional intensity by peak-fitting following Penttilä et al. (2020).^32^ An instrumental broadening of 0.02 Å^–1^ was determined from a LaB_6_ sample and taken into account when calculating the crystallite size. A crude estimate for the sample crystallinity index was obtained by dividing the integrated contribution of the equatorial crystalline peaks by the total area under the equatorial intensity curve on the *q* range from 0.5 to 2.25 Å^–1^.

## Results and Discussion

**Figure 2:**
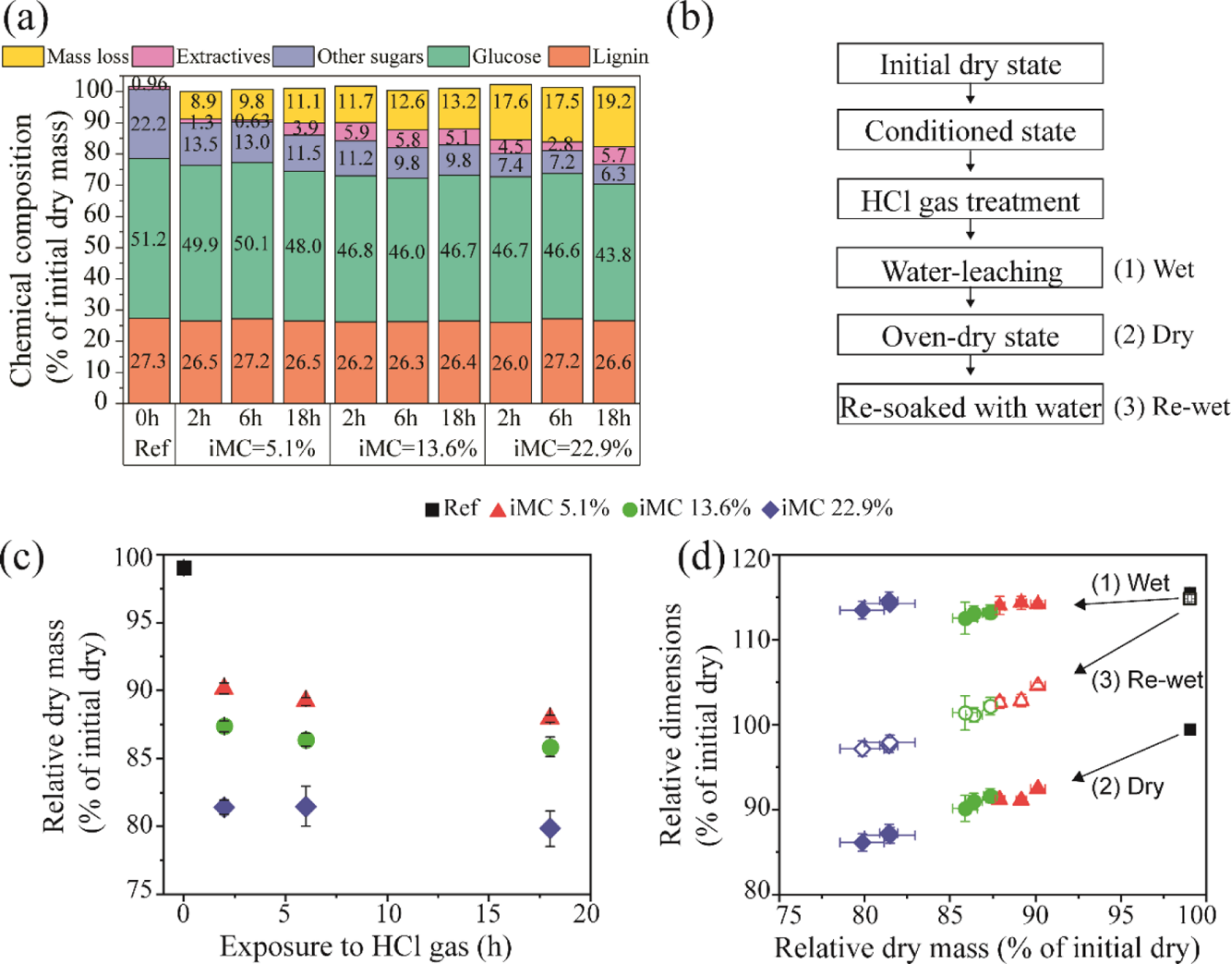
Changes in chemical composition, mass, and dimensions: (a) chemical composition of the wood with different iMCs and after different HCl gas exposure times. The calculations are based on the initial dry mass (before the HCl gas treatment). Ash content <0.5% is omitted in the figure; (b) treatment sequence and geometry of wood blocks; (c) relative dry mass in dependence on the exposure to HCl gas; and (d) relative dry and wet dimensions (radial × tangential) depending on the relative dry mass. The relative dry mass in (c) of the reference sample below 100% is explained by the leaching of native extractives from wood during the HCl treatment.

The chemical composition presented in Figure 2a is given as the percentage of the initial dry mass before the treatment. The mass loss is solely from a loss in cell wall polysaccharides (cellulose or hemicelluloses), as can be seen from the lower quantities of glucose and other sugars, in contrast to the constant lignin content.

It is worth noticing that common processes such as thermal modification^27,33^ and hydrothermal treatment^34,35^—in which hemicelluloses are removed—present formation of the so-called “pseudo-lignin”. These lignin-like structures, despite originating from polysaccharides degradation, are quantified as Klason lignin.^36^ Herein, although an acid system is applied, there is no indication for the formation of pseudo-lignin, as lignin content remained nearly unchanged. Therefore, HCl gas treatment shows a clear advantage over other biomass pretreatments, as pseudo-lignin has detrimental effects on biological conversion and cellulosic fiber production of pretreated biomass.^37^

Moreover, a clear degradation of hemicelluloses can be observed in Figure 2a by the percentage of sugars other than glucose, which decreased from 22% in the untreated wood to ca. 6% after the harshest treatment (iMC of 22.9% at 18 h). The removal of hemicellulose during pretreatment of lignocellulosic biomass can be a potential side stream to generate sugars and fuels. Among the three major components of wood, hemicellulose indeed is the polymer which degrades most easily under a variety of conditions ranging from acidic conditions as in hydrothermal treatment,^34,35,38^ to alkaline as kraft process^39^ and thermal modifications of wood.^33,40^ This is due to the irregular, noncrystalline structure of hemicelluloses and consequently a more open structure with a lower degree of polymerization and higher hygroscopicity compared to that of cellulose.

Both hemicellulose and cellulose show less distinction in content among different times of exposure to HCl and the same iMC group, highlighting the greater effect of iMC rather than exposure time on the degree of hydrolysis. The exposure time may, however, have an effect at shorter reaction times, which were not studied here.

The physical changes in the HCl gas-treated samples were determined on wood blocks following the steps as shown in Figure 2b. All samples showed an accentuated mass loss in the first 2 h of HCl gas treatment (Figure 2c). Longer HCl treatment times caused only a small additional mass loss in the different iMC groups. Regardless of the HCl treatment time, a higher iMC resulted in higher dry mass loss. The initial moisture in the wood sample seemed to determine the amount of HCl gas adsorbed onto the solid material, leading to more severe hydrolysis at a higher iMC of wood samples.

Figure 2d shows a correlation of the relative dimensions in different states [wet after the HCl treatment (1), oven-dry (2), and re-wet after resoaking in water (3)] with the relative dry mass. The relative wet (water-saturated) dimensions after the HCl treatment remained unchanged, presumably because water molecules occupied the space that was previously occupied by hydrolyzed cell wall constituents. When the water was removed from the samples during oven drying, the cell walls shrank, and the relative dry dimensions decreased as a linear function of the relative dry mass. This is an indication that the additional cell wall space that was created by the removal of cell wall constituents collapsed during oven drying. Resoaking the oven-dried samples in deionized water resulted in the swelling of the samples, but the initial wet dimensions of the samples were not recovered completely. The difference between the relative wet dimensions after the HCl treatments and those after drying and resoaking increased with decreasing relative dry dimensions. Presumably, once the cell wall space that was created by the removal of wood constituents (Figure 2a) collapsed, it could not be reopened by full water saturation. The same irreversible loss in water-accessible cell wall space also occurs upon drying of pulp fibers, pressurized hot water extracted wood, and untreated, freshly felled green wood upon drying.^2,27,41^

**Figure 3:**
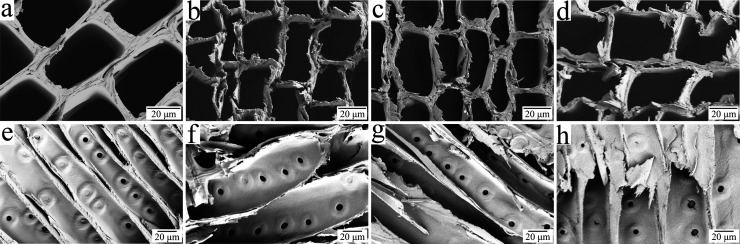
SEM images of the original wood and HCl gas-treated samples. Cross-section (a–d) and radial section (e–h). The results are shown for reference samples (a,e) and samples treated with HCl gas for 18 h, with iMC 5.1 (b,f), 13.6 (c,g), and 22.9% (d,h).

The scanning electron microscopy (SEM) images of samples treated with HCl gas for 18 h (Figure 3b–d,f–g) show a well-preserved cellular structure compared to the reference wood (Figure 3a,e). These observations were rather similar between the different iMC samples. On the cross-section, the cell walls in the HCl-treated samples appeared somewhat distorted and broken (Figure 3b–d). Additional images on samples after HCl treatments at 5.1% iMC showed that these defects are less evident at the mildest treatment (iMC 5.1% at 2 h) (Figure S1). HCl treatments of cellulosic samples can lead to visible fiber cleavage and degradation, particularly after extended reaction times.^15^ Nonetheless, we point out that the observed defects in the wood could be partly caused by the SEM sample preparation due to the fragility of the HCl-treated samples. In fact, in the radial sections, it is possible to confirm that intact-appearing cell walls with bordered pits could still be observed in the HCl-treated samples (Figure 3f,g), despite the harsh hydrolysis conditions.

**Figure 4:**
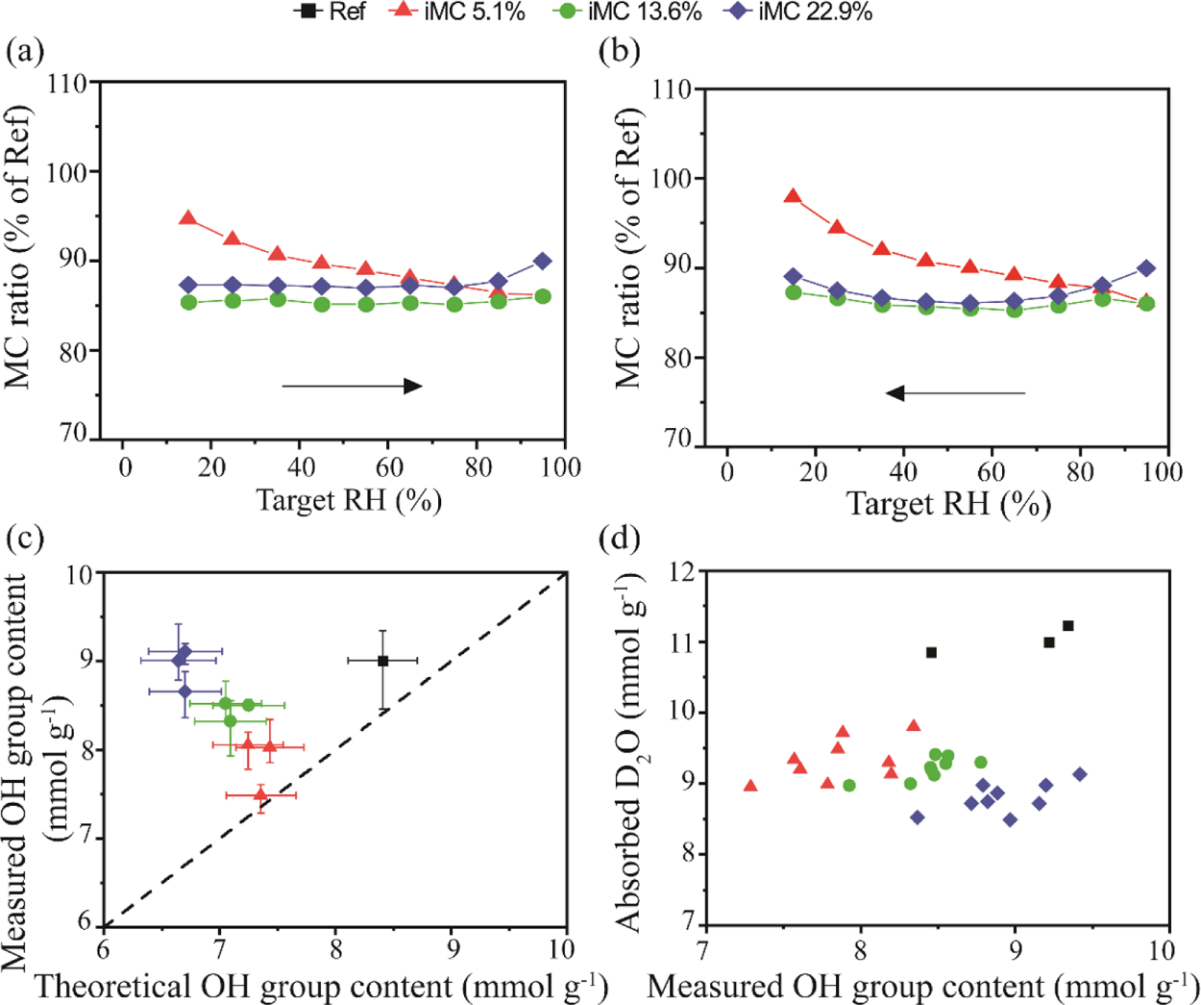
Results of the dynamic water vapor sorption measurements in the samples treated in HCl gas for 18 h: MC ratios in dependence on the RH based on measurements in absorption (a) and (scanning) desorption (b). Measured accessible OH group content in dependence on the theoretical accessible OH group content (c) and amount of absorbed D_2_O in dependence on the accessible OH group content (d). The data points in (c) represent average values, and the error bars show the data range.

The interaction of the treated wood with water vapor was analyzed using an automated sorption balance in the range between 0 and 95% RH. The sorption balance allows a precise control of temperature (±0.2 °C) and RH as well as an accurate mass determination (±0.1 μg), but the measured MC at the end of each RH step may slightly deviate from the true equilibrium MC depending on the hold times (d*m*/d*t*) chosen.^42^ Besides the untreated reference, samples that were treated in HCl vapor for 18 h at different iMCs were analyzed (Figure 4).

The HCl gas treatment reduced the moisture uptake of the wood across the hygroscopic range, as can be seen by the lower absorption and scanning desorption isotherms of the treated woods compared to the reference (Figure S2). However, the difference in MC between the samples that were treated at different iMCs was small, despite the large differences in the relative dry mass and the residual carbohydrate contents. The MC differences were further analyzed by calculating MC ratios, which relate the MC of the treated wood to the corresponding MC of the reference at each RH step (Figure 4a,b). The MC ratios revealed that the effectiveness at which the different treatments reduced the MC of the wood depended on the RH level. The treatment at low iMC (5.1%) resulted in a decreasing MC ratio with increasing RH, while the MC ratio of the other treatments either remained constant (iMC 13.6%) or even increased slightly (iMC 22.9%). At 95% RH, treatments at low and intermediate iMC had nearly identical MC ratios of ca. 86%, while the HCl treatment at high iMC resulted in an MC ratio of ca. 90%.

These results show that the change in sorption behavior was not only affected by the loss in hydrophilic hemicelluloses but also by other factors. A similar effect was found for treatments of wood at elevated temperatures, where higher iMCs during the treatment not only facilitated wood hydrolysis but also resulted in a less efficient reduction of the water vapor sorption. It was speculated that the cleavage of covalent bonds in the wood residue by strong hydrolysis reduced the cell wall matrix stiffness and facilitated the opening of the cell wall to accommodate water molecules.^43^ However, in view of the possible limitation in attaining a true equilibrium MC during short holding times, the MC ratio across the measured RH range between the samples may have been influenced by differences in the sorption rate. The increasing MC ratio of samples treated at high iMC could have resulted from an increase in sorption rate compared to the reference, which possibly reduced the deviation between measured and equilibrium MC. The opposite may apply to samples that showed a decreasing MC ratio. Hence, the observed sample-to-sample variation in water vapor sorption may not only originate from differences in the thermodynamic equilibrium that the samples approached at each RH step but possibly also from differences in the time required to reach this equilibrium state.

The measured, accessible OH content was determined based on the dry mass increase caused by deuterium exchange of OH to OD (Figure 4c) and is presented as a function of the theoretical, accessible OH group content, which is calculated based on the samples’ chemical composition (Figure 2a), following reference values.^28,29^ For the reference sample, we measured an average accessible OH group content of 9 mmol g^–1^, which is in line with the contents of 8.4 and 9.3 mmol g^–1^ reported by Thybring et al. (2017)^29^ for air-dried Norway spruce earlywood and latewood, respectively. However, this measured accessible OH group content exceeded the theoretical content of ca. 8.4 mmol g^–1^ that we calculated on the basis of the chemical composition. Besides possible errors in the chemical composition analysis and in the assumed constants for the calculation of the theoretical accessible OH group content, the applied deuterium exchange approach may overestimate the actual OH accessibility. Such an overestimation may be caused by residual amounts of absorbed water at the end of the initial and final drying steps of the deuterium exchange protocol. Thereby, the sample mass may have been additionally increased by an exchange of residual H_2_O with residual D_2_O molecules and not only by the exchange of accessible OH groups.

Despite a possible overestimation by the deuterium exchange method, we would have expected the preferential removal of hemicelluloses by the HCl treatment to reduce the accessible OH group content. A reduction in accessible OH group content by the removal of OH-rich hemicelluloses (and most likely some parts of the cellulose) has been shown previously for heat-treated wood.^44^ HCl treatments at low iMC (5.1%) indeed reduced the measured, accessible OH group content compared to the reference (Figure 4c). However, increasing the iMC of the HCl treatment resulted in an increase in the accessible OH group content despite the additional loss in hemicelluloses. This effect led to the progressive deviation between measured and theoretical accessible OH group content. This deviation could have been caused by a larger overestimation of accessible OH groups in the treated samples. However, we do not see any obvious reason why the residual amounts of water molecules during the drying steps of deuterium exchange measurement increased for the treated samples, particularly because their sorption isotherms showed a reduced water absorption. Instead, our results suggest that the changes in the accessible OH group content of the treated samples were unrelated to chemical composition changes and/or influenced by other factors. Even if our calculation of the theoretical OH group content was invalid, it is still remarkable that a strong HCl treatment (e.g., iMC 22.9%, duration 18 h) removed considerable proportions of hemicelluloses without changing the accessible OH group content compared to the reference sample. We may speculate that this could be caused by small changes in the molecular organization at the microfibril surfaces, which would influence both the validity of the assumptions used for calculating the theoretical accessibility of OH groups and the actual accessible OH group content.

We also determined the concentration of D_2_O molecules that were absorbed to each OH group in the wood at the end of the exposure to D_2_O vapor, and this is shown as a function of the measured concentration of accessible OH groups in Figure 4d. In agreement with recent studies,^27,45−47^ the number of accessible OH groups was not the main factor in controlling the concentration of absorbed water molecules in wood. HCl gas treatments reduced the concentration of absorbed D_2_O molecules compared with the reference, but this reduction was unrelated to changes in the accessible OH group concentration. This was particularly noticeable after HCl treatments at high iMC of wood that resulted in a reduction in the concentration of absorbed D_2_O from ca. 11 to <9 mmol g^–1^ despite no change in the accessible OH group concentration. Leboucher et al. (2020)^20^ reported similar results for cellulose hydrolyzed in HCl vapor using high-temperature FT-IR spectroscopy on deuterated samples. They found less bound water in hydrolyzed cellulose but a larger ratio of exchanged OH groups compared to nonhydrolyzed cellulose once the samples were heated.

**Figure 5:**
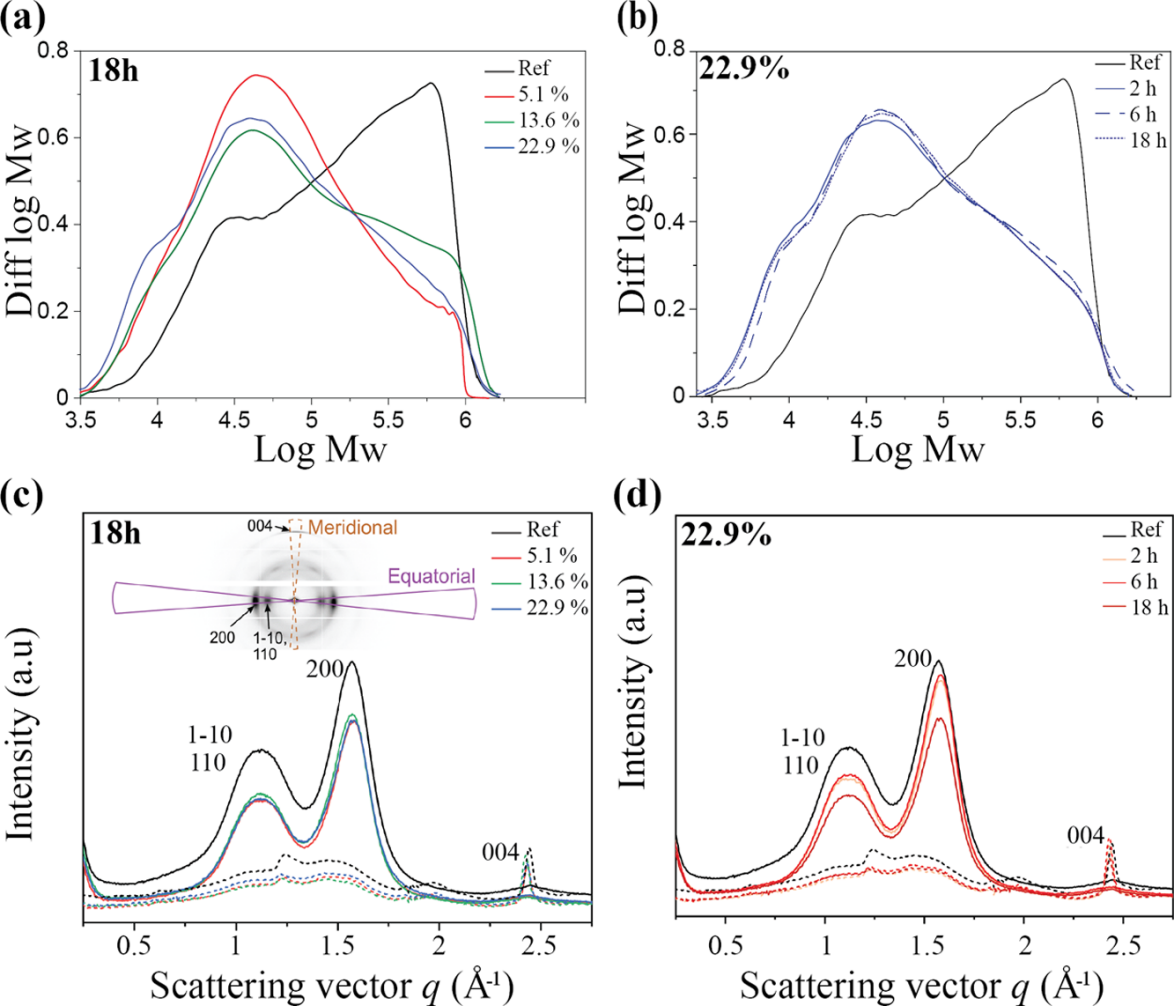
Molecular mass distributions of holocellulose from reference and HCl gas-treated wood at 18 h of HCl exposure differing in iMC (a) and at an iMC of 5.1% differing in time of exposure to HCl gas (b), determined by GPC. WAXS intensities of reference and HCl gas treated wood at 18 h of HCl exposure differing in iMC (c) and at an iMC of 22.9% differing in time of exposure to HCl gas (d), integrated over equatorial (solid line) and meridional (dashed line) sectors as illustrated in the two-dimensional scattering image (inset in c).

The holocellulose portion from reference and HCl gas-treated samples was analyzed for its molar mass distribution. A decrease in the molecular mass is clearly seen with all HCl-treated wood samples when compared to the untreated wood sample. Among the treated samples, the portion of low molecular mass polymers is the highest with the highest iMC, as presented in Figure 5a for samples exposed to HCl gas for 18 h. This trend can also be observed, to a lower extent, for 2 and 6 h of exposure time to HCl (Figure S3c,d). Acid-catalyzed hydrolysis is dependent on the amount of water which dissociates HCl. Thus, samples treated with higher iMC (22.9%) resulted in a higher number of chain scissions per cellulose chain which denotes lower molecular mass.^16^

Although all HCl-treated samples showed a clear decrease in molecular mass compared to the untreated wood, the effect of the exposure time in each iMC group was unclear. For samples treated at 5.1% iMC, prolonging the exposure time to HCl gas led to a further decrease in molecular mass (Figure S3a). The opposite was observed for samples treated at 13.6% iMC (Figure S3b), while the exposure time had no effect on samples with 22.9% iMC (Figure 5b). The shift in the molar mass distribution to lower molecular weight (*M*_W_) with the acid hydrolysis is explained by the chain scissions of cellulose and hemicelluloses. However, within harsh acid hydrolysis conditions (22.9% iMC), the molar mass distribution is quite independent of the hydrolysis time, which means that the leveling-off degree of polymerization (LODP) has been reached. The low *M*_W_ peak in hydrolyzed samples settles around 40,000 g mol^–1^ (DP 250), which is higher than the LODP values generally reported for wood-based substrates (100–200).^18^ Although hemicelluloses in softwoods and hardwoods are different, we note that the LODP value of 250 is similar to hardwood-based pulp substrates with high xylan content, where xylan has been speculated to protect the cellulose from reaching lower LODP values.^48^ The small shoulder around 10,000 g mol^–1^ (DP 60) in the hydrolyzed samples coincides both with a proposed recalcitrant fraction of xylan in hardwood pulps^48^ and the low-*M*_W_ fraction of cellulose hydrolyzed down to LODP.^18,21^

The WAXS intensities (Figures 5c,d,and S4) did not show any major differences due to the HCl gas hydrolysis, indicating that the crystalline cellulose microfibrils were not largely modified by the treatment. As the only clear change, the overall intensity level of the reference sample was noticed to be higher as compared to the hydrolyzed ones. This was particularly evident in the *q* range 0.3–0.8 Å^–1^, where no strong diffraction peaks from cellulose appear. The difference is most likely due to the removal of the less-ordered component consisting of mainly hemicelluloses and lignin (Figure 2a), which contribute to the intensities on a broader *q* range than crystalline cellulose.^49^ The parameters resulting from peak fitting (Table S1) were rather similar between the different samples. In general, smaller values of the lattice spacing of (200) planes (3.97–4.00 Å) and larger values of the lattice spacing of (004) planes (2.57–2.59 Å), and the crystallite width (3.0–3.2 nm) and length (18–21 nm) were observed for the hydrolyzed samples, but no clear correlation with the severity of the treatment could be found. Similar minor differences due to HCl gas or vapor hydrolysis have been detected in previous X-ray diffraction experiments,^16,18^ and, for instance—the decrease of the 200 lattice spacing agrees with simulations of recrystallizing cellulose chains after cleavage.^19^ Most importantly, however, the crystallinity index increased clearly (by 10–20%), which is a common result obtained for various cellulosic substrates subjected to HCl gas or vapor hydrolysis.^16−18^ In the current case, it can be assigned to the removal of noncrystalline material and the small increase in crystallite dimensions. Although the simple peak-fitting method applied in the current work does not necessarily yield the true crystalline fraction in the sample, the observed change is expected to correspond to a real increase in crystallinity, which is also visible as a decrease of the broad, noncrystalline scattering contribution underlying the diffraction peaks in Figure 5c,d.

## Conclusions

In this work, we applied HCl gas to wood at different iMCs, in order to manipulate the deconstruction of the wood macrostructure and to understand the effect on its microstructure. SEM images revealed a preserved anatomical structure of the wood even with harsher HCl gas treatments. Differences in the iMC of wood prior to HCl treatment played a key role in hydrolysis rather than the time of exposure to the acidic gas. In particular, a higher iMC correlated with a higher degradation of hemicelluloses. On the other hand, no major differences were observed in the crystalline cellulose microfibrils due to the treatments. Remarkably, the hydrogen–deuterium exchange technique showed a contradictory increase in the accessible OH group concentration after treatments at high iMC, despite additional removal of hemicelluloses. Overall, HCl treatments showed to be an effective method for a controlled deconstruction of wood biomass, particularly regarding the extraction of hemicelluloses while preserving cellulose and lignin moieties in an intact wood cell wall.

## Supporting Information Available

SEM images of the original wood and HCl gas-treated samples; results of the DVS measurements in the samples treated in HCl gas for 18 h; molar mass distributions of holocellulose samples determined by GPC; equatorial and meridional WAXS intensities of reference and HCl gas-hydrolyzed wood; and results from WAXS analysis of reference and HCl gas-hydrolyzed wood (PDF)
