# Supplementary material for: Effect of Moisture on Polymer Deconstruction in HCl Gas Hydrolysis of Wood
Source: ACS Omega. 2022 Feb 16;7(8):7074–83. doi: 10.1021/acsomega.1c06773 (PMC8892909; doi:10.1021/acsomega.1c06773)
Supplement: Supplementary file 1 — ao1c06773_si_001.pdf [file ao1c06773_si_001.pdf]

Supporting Information (SI)

Effect of moisture on polymer deconstruction in HCl gas hydrolysis of wood

Tainise Lourençon[1], Michael Altgen[1,2*], Timo Pääkkönen[1], Valentina Guccini[1], Paavo Penttilä[1*], Eero Kontturi[1], Lauri Rautkari[1*]

[1] Department of Bioproducts and Biosystems, Aalto University, P.O. Box 16300, FI-00076 Aalto, Finland
[2] Universität Hamburg, Department of Biology, Institute of Wood Science, Leuschnerstraße 91c, DE-21031 Hamburg, Germany

*corresponding authors: michael.altgen@uni-hamburg.de; paavo.penttila@aalto.fi; lauri.rautkari@aalto.fi

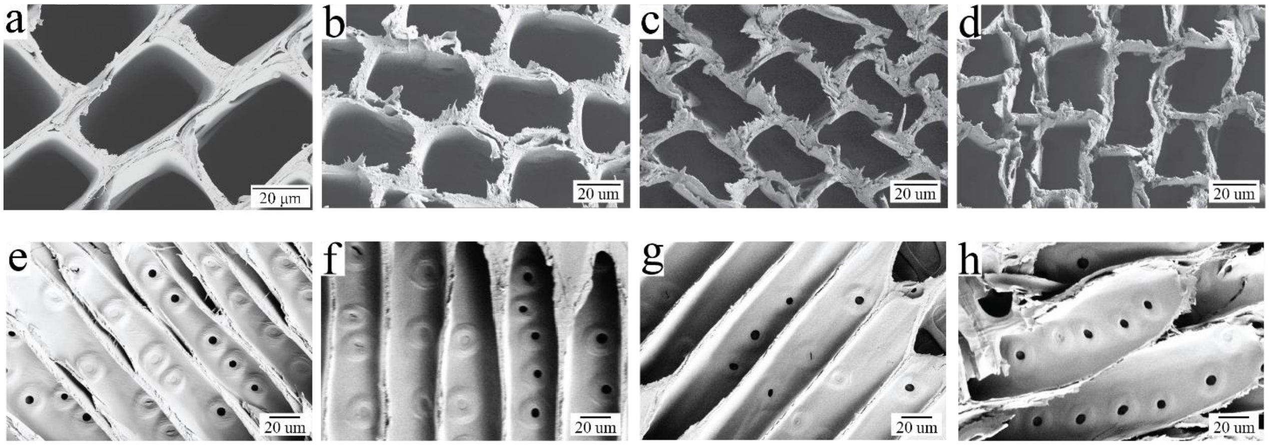

Figure S1. SEM images of original wood and HCl gas treated samples. Cross-section (a-d) and radial section (e-h). The results are shown for reference samples (a, e) and samples treated with HCl gas at iMC 5.1% at exposure time of 2 h (b, f), 6 h (c, g) and 18 h (d, h).

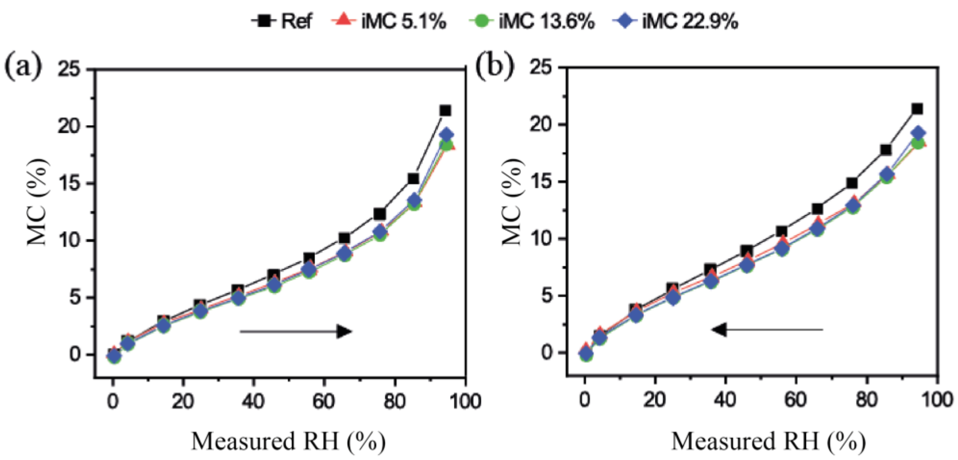

Figure S2. Results of the dynamic water vapor sorption measurements in the samples treated in HCl gas for 18 h: Absorption (a) and (scanning) desorption isotherms (b).

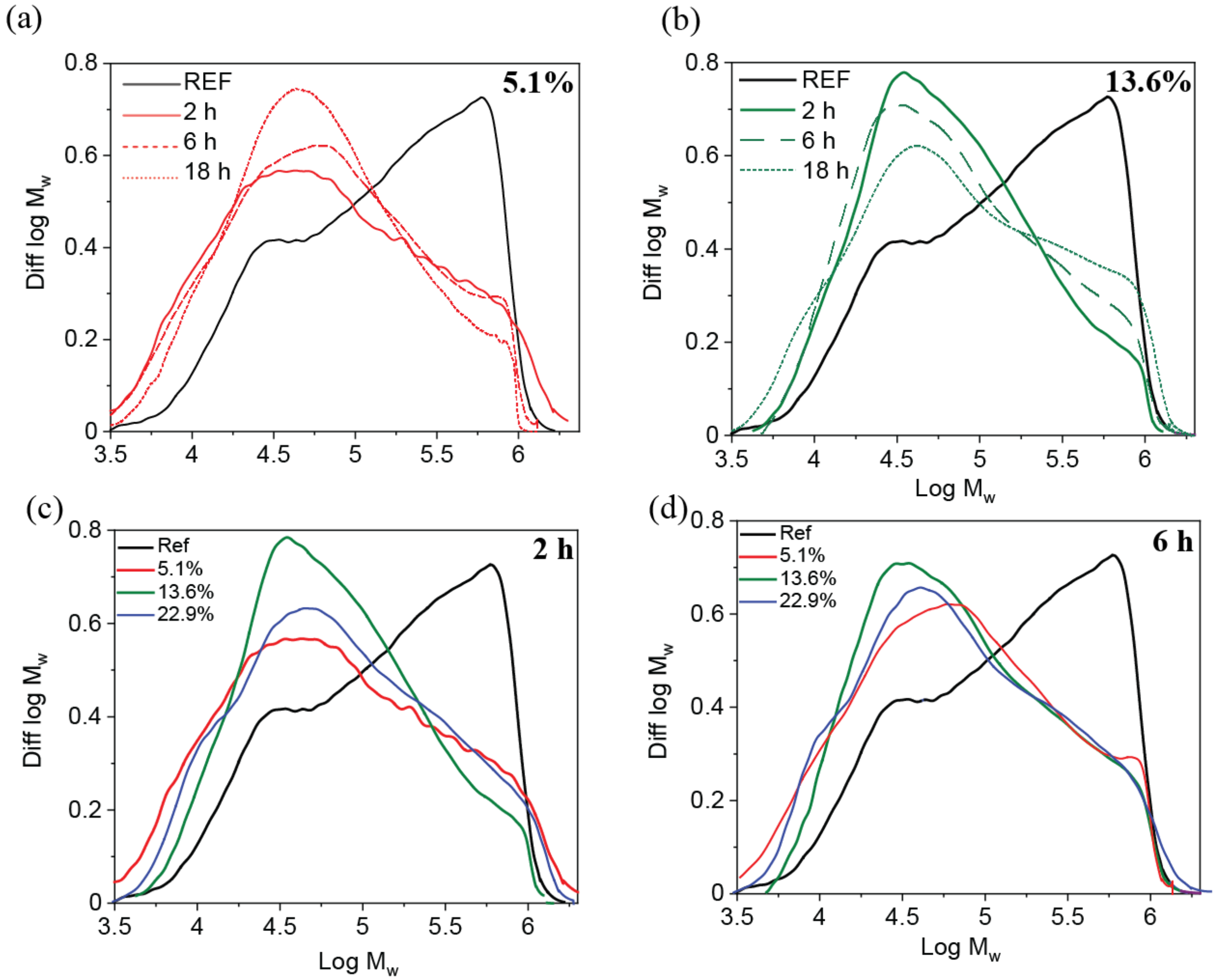

Figure S3. Molecular mass distributions (determined by GPC) of holocellulose samples at iMC 5.1% (a) and iMC 13.6% (b) treated with different exposure times to HCl gas and samples exposed to HCl gas for 2 h (c) and 6 h (d) differing in iMC.

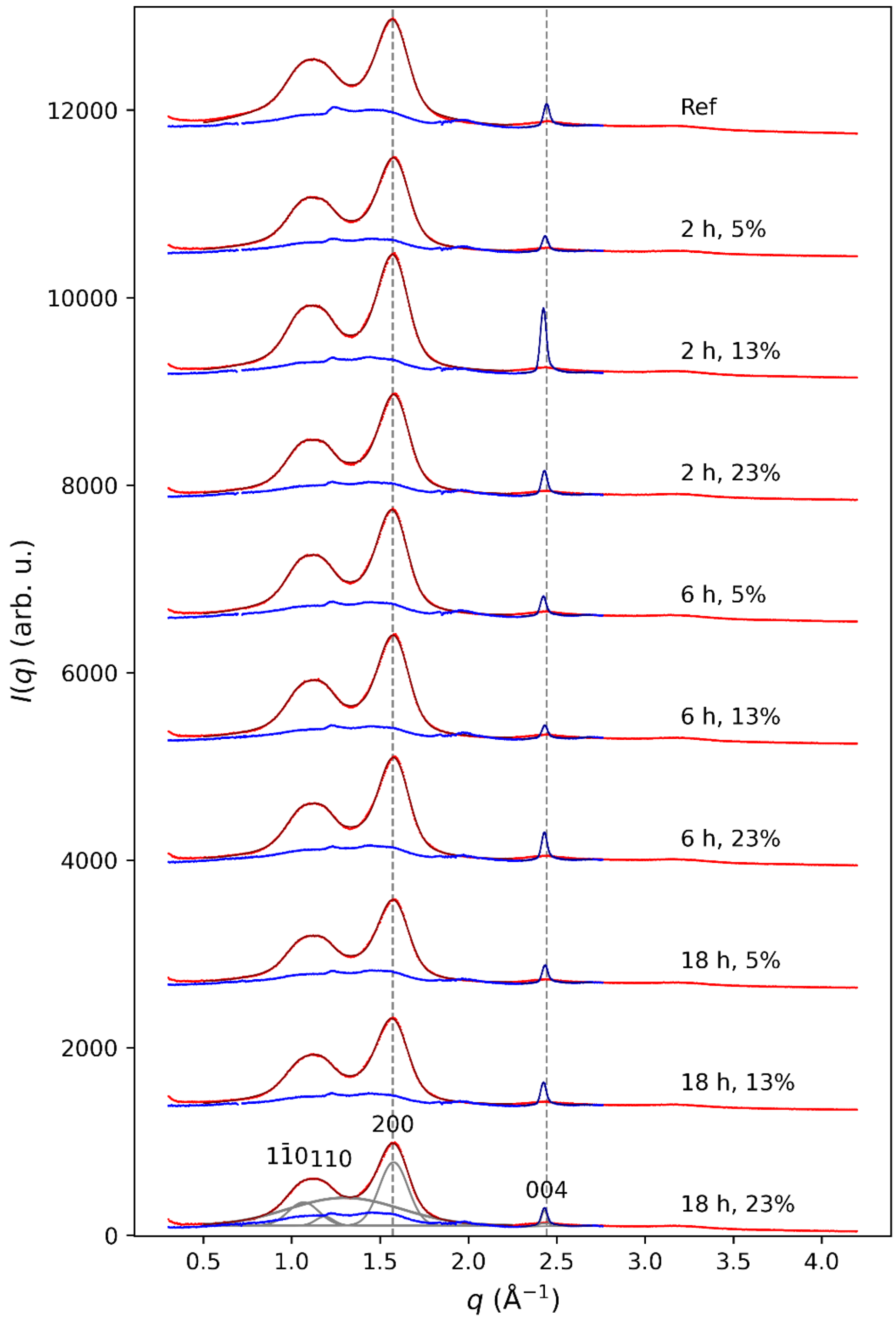

Figure S4. Equatorial (red) and meridional (blue) WAXS intensities of reference and HCl gas hydrolyzed wood, with dots corresponding to data points and solid lines to fits. The different components of the fits are shown by grey solid lines in the lowermost data, and the locations of reflections 200 and 004 in the reference sample are indicated by grey dashed lines to illustrate the shifts of the peaks.

Table S1. Results from WAXS analysis of reference and HCl gas hydrolyzed wood

	Lattice spacing d_{200} (Å)	Crystal width L_{200} (nm)	Lattice spacing d_{004} (Å)	Crystal length L_{004} (nm)	Crystallinity index
Reference	4.00	3.05	2.57	19	0.31
2 h, 5%	3.98	3.11	2.58	19	0.36
2 h, 13%	3.99	3.10	2.59	18	0.38
2 h, 23%	3.97	3.17	2.59	20	0.37
6 h, 5%	4.00	3.10	2.59	20	0.37
6 h, 13%	3.98	3.06	2.58	21	0.38
6 h, 23%	3.98	3.14	2.59	21	0.37
18 h, 5%	3.98	3.10	2.58	21	0.36
18 h, 13%	4.00	3.00	2.59	20	0.37
18 h, 23%	3.98	3.09	2.59	19	0.33
